# Intranasal hemagglutinin protein boosters induce protective mucosal immunity against influenza A viruses in mice

**DOI:** 10.1073/pnas.2422171122

**Published:** 2025-09-24

**Authors:** Miyu Moriyama, Gisele Rodrigues, Jiping Wang, Radeesha Jayewickreme, Andrew Hudak, Huiping Dong, Robert J. Homer, Shuangge Ma, Akiko Iwasaki

**Affiliations:** ^a^Department of Immunobiology, Yale School of Medicine, New Haven, CT 06510; ^b^Center for Infection and Immunity, Yale School of Medicine, New Haven, CT 06510; ^c^Department of Biostatistics, Yale School of Public Health, New Haven, CT 06510; ^d^Department of Pathology, Yale University School of Medicine, New Haven, CT 06510; ^e^Department of Molecular Cellular and Developmental Biology, Yale University, New Haven CT 06511; ^f^HHMI, Chevy Chase, MD 20815

**Keywords:** viruses, respiratory, vaccine, mucosal, antibodies

## Abstract

Licensed parenteral influenza vaccines induce systemic antibody responses and alleviate disease severity but do not efficiently induce local mucosal immune responses. Here, we describe an intranasal booster strategy with unadjuvanted recombinant hemagglutinin (HA) following initial messenger RNA-lipid nanoparticle (mRNA-LNP) vaccination, Prime and HA. This regimen establishes highly protective HA-specific mucosal immune memory responses in the respiratory tract. Intranasal HA boosters resulted in significantly reduced viral replication compared to parenteral mRNA-LNP boosters in both young and old mice. Correlation analysis revealed that slightly increased levels of nasal Immunoglobulin A (IgA) are significantly associated with a reduced viral burden in the upper respiratory tract. Intranasal boosting with bivalent H1 HA induced mucosal immunity against vaccine-matched and mismatched heterologous influenza viruses. Additionally, a heterosubtypic intranasal H5 HA booster elicited H5-reactive mucosal humoral responses in H1-surviving mice. Our work illustrates the potential of a nasal HA protein booster as an adjuvant-free strategy to prevent infection and disease from influenza A viruses.

## Significance

While current influenza vaccines protect against severe diseases, shortcomings include limited cross-protection, short-lived immunity, and vaccine hesitancy concerns including the fear of needles. We describe a nasal booster approach, Prime and hemagglutinin (HA), with unadjuvanted recombinant HA antigens that addresses key challenges in influenza vaccination by inducing strong local immunity against multiple influenza strains in mice. By stimulating localized mucosal defenses, including Immunoglobulin A (IgA), Immunoglobulin G (IgG), and tissue-resident memory cells, Prime and HA established a frontline barrier against infection at the point of viral infection. This needle-free delivery method for the booster doses has the potential to enhance vaccine uptake to increase vaccine coverage in the vulnerable population.

Seasonal influenza epidemics present a significant public health challenge, leading to approximately half a million deaths globally each year (1). Despite vaccines being the most effective preventive measure against seasonal influenza, their effectiveness remains limited, ranging between 19% and 54% (Centers for Disease Control and Prevention. Seasonal influenza vaccine effectiveness studies, 2014–2024. https://www.cdc.gov/flu/professionals/vaccination/effectiveness-studies.htm. Accessed 13 July 2024). Furthermore, recent data estimate that the effectiveness of seasonal influenza vaccines in preventing secondary influenza A infections among household contacts is as low as 5% (2), highlighting the need for improving influenza vaccine performance.

One promising avenue to enhance vaccine effectiveness is to improve mucosal immune responses alongside systemic immunity. Conventional inactivated split influenza vaccines are administered intramuscularly and are insufficient at inducing robust mucosal immune responses including IgA and tissue-resident memory cells in the respiratory mucosa, which are crucial for rapid anamnestic responses that block the spread of respiratory viruses (3, 4). The development of vaccines capable of inducing such protective local immune responses may offer better protection against disease, infection, and transmission of influenza and other respiratory viruses (5).

Recent studies have shown that vaccine regimens combining parenteral priming with intranasal boosting can induce robust mucosal immune responses and enhance protection against viral challenges, as demonstrated in severe acute respiratory syndrome coronavirus-2 (SARS-CoV-2) animal models (6–8). In a previous study, we introduced the “Prime and Spike” strategy, in which intramuscular priming with a Spike-encoding mRNA-LNP vaccine (prime) was followed by intranasal boosting with an unadjuvanted, unformulated, trimeric Spike protein (Spike). This approach generated strong protective mucosal immune responses and reduced viral transmission (7).

Building on these findings, this report explores the use of the hemagglutinin (HA) mRNA-LNP vaccine platform for priming and demonstrates that intranasal unadjuvanted HA protein boosters, termed “Prime and HA,” can evoke robust clinical and virological protection against respiratory viral challenge. As multiple clinical trials are underway to test the efficacy of influenza mRNA vaccines (9), our study highlights the potential of the “Prime and HA” strategy to enhance influenza vaccine effectiveness and contribute to better control of seasonal influenza epidemics and future pandemics.

## Results

### Intranasal Unadjuvanted HA Protein Boosters Confer Robust Protection in Mice Primed with Intramuscular mRNA-LNP Vaccination.

**Fig. 1.**
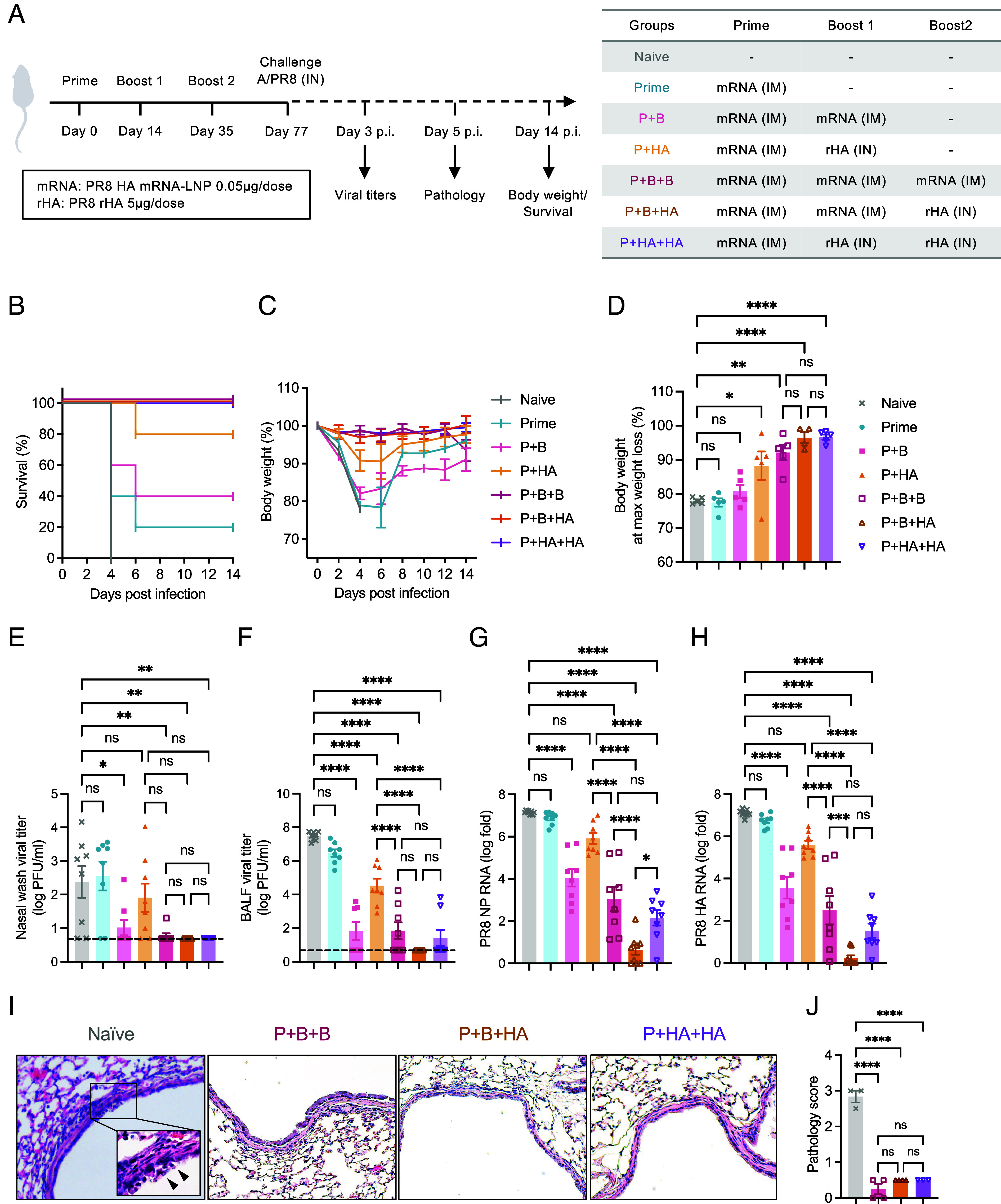
Intranasal unadjuvanted HA protein boosters following parenteral mRNA-LNP immunization provide robust protection. (*A*) Balb/c mice were immunized with 0.05 μg of PR8 HA mRNA-LNP intramuscularly and 5 μg recombinant PR8 HA protein intranasally as indicated. Six weeks after the third dose, mice were challenged with 10^4^ PFU of A/PR8. (*B*–*D*) Survival and body weight were monitored for 14 d. (*E*–*H*) Nasal wash, BALF, and lung tissue were collected 3 d after infection, and infectious viral titers in nasal wash (*E*) and BALF (*F*) were determined by the plaque assay. Viral RNA levels in the lung tissue were determined by RT-qPCR using primers targeting the PR8 NP gene (*G*) and PR8 HA gene (*H*). (*I* and *J*) The immunized Balb/c mice were challenged with 10^5^ PFU of A/PR8. Lung tissue was collected 5 d after infection for pathological assessment by H&E staining. Morphologic cell death and degeneration are observed in the naive lung (arrowheads in the *Inset*). Data are representative or pooled from at least two independent experiments. Error bars are shown in mean ± SEM. Statistical significance was tested using one-way ANOVA with Tukey’s multiple comparison test. ns, nonsignificant; *P < 0.05; **P < 0.01; ***P < 0.001; ****P < 0.0001.

To evaluate the protective efficacy of the parenteral priming followed by intranasal boosting strategy against lethal influenza virus infection, we immunized Balb/c mice intramuscularly (IM) with an mRNA-LNP vaccine encoding influenza A/PR8 HA (0.05 μg per dose) and boosted them with intranasal (IN) unadjuvanted recombinant A/PR8 HA protein (5 μg per dose), followed by A/PR8 challenge (Fig. 1*A*). A subprotective dose (0.05 μg) of A/PR8 HA mRNA-LNP for IM priming protected 20% of the mice from death and did not prevent severe weight loss (Fig. 1 *B*–*D*) and a high viral load in both the upper and lower respiratory tracts (Fig. 1 *E*–*H*). A single IN A/PR8 HA protein booster (Prime and HA; P+HA) substantially mitigated weight loss (Fig. 1 *C* and *D*) but was still insufficient to fully protect mice from death (Fig. 1*B*) and viral replication (Fig. 1 *E*–*H*). In contrast, an IN A/PR8 HA protein booster following either two doses of IM A/PR8 HA mRNA-LNP (Prime and boost and HA; P+B+HA) or P+HA (Prime and HA and HA; P+HA+HA) completely protected mice from morbidity and mortality (Fig. 1 *B*–*D*) and minimized lung tissue pathology (Fig. 1 *I* and *J*) to levels comparable to those with three doses of A/PR8 HA mRNA-LNP (Prime and boost and boost; P+B+B). Notably, IN A/PR8 HA protein booster (P+B+HA) conferred robust protective immunity, as evidenced by the absence of infectious virus in the nasal wash and bronchoalveolar lavage fluid (BALF) (Fig. 1 *E* and *F*) and only minimal viral RNA remnants in the lungs (Fig. 1 *G* and *H*), a level of protection not achieved by the parenteral A/PR8 HA mRNA-LNP booster (P+B+B). Although less efficient than P+B+HA, animals that received P+HA+HA displayed reduced viral replication compared with P+HA (Fig. 1 *E*–*H*), highlighting the importance of repeated IN A/PR8 HA protein administration following A/PR8 HA mRNA-LNP priming.

We next assessed the efficacy of the vaccine strategy against a different influenza virus strain over an extended period postimmunization. The protective immunity was robustly induced following P+B+HA and P+HA+HA against another H1N1 strain, A/Michigan/45/2015 (MI15), and was sustained for at least 12 wk after the second boost (*SI Appendix*, Fig. S1 *A*–*F*). Conversely, a single IM immunization with a quadrivalent influenza vaccine (QIV) containing 0.75 μg of MI15 HA, the current standard of care, only partially protected mice from weight loss and did not substantially reduce the viral load at this time point (*SI Appendix*, Fig. S1 *A*–*E*). Further, IN MI15 HA booster given to QIV-primed mice failed to confer antiviral immunity. These results underscore the superior ability of IN HA protein boosters to block infection in the nose and lung over parenteral mRNA-LNP boosters following the initial series of IM mRNA-LNP vaccination, but not after QIV.

### P+B+HA and P+HA+HA Establish Mucosal Immune Memory Responses.

**Fig. 2.**
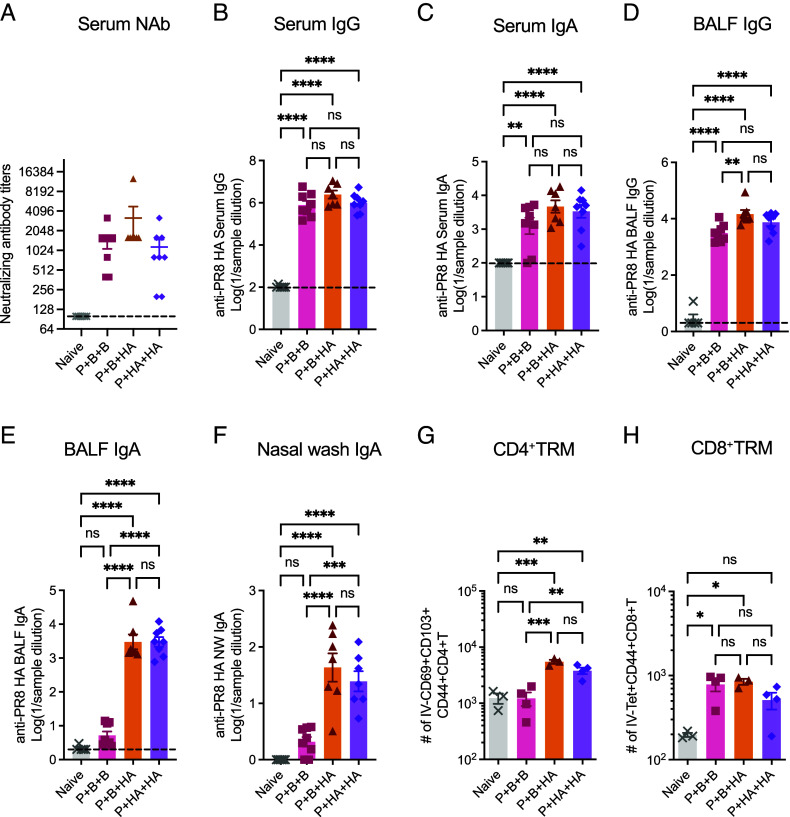
Intranasal HA boosters following mRNA-LNP immunizations establish mucosal humoral and cellular immune responses. Balb/c mice were primed and boosted with 0.05 μg of PR8 HA mRNA-LNP intramuscularly and received either an additional dose of PR8 HA mRNA-LNP (Prime and boost and boost; P+B+B) or 5 μg recombinant PR8 HA protein intranasally (Prime and boost and HA; P+B+HA). Another group of Balb/c mice was primed with 0.05 μg of PR8 HA mRNA-LNP intramuscularly and received two doses of 5 μg recombinant PR8 HA booster intranasally (Prime and HA and HA; P+HA+HA). Six weeks after the second boost, serum, BALF, nasal wash, and lung tissue were corrected and assessed for humoral (*A*–*F*) and cellular (*G* and *H*) immune responses. (*A*) Serum neutralizing antibody titers against A/PR8 strain were determined by the microneutralization assay. (*B*–*F*) PR8 HA-specific systemic and mucosal IgG and IgA responses were measured by ELISA. (*G* and *H*) Polyclonal CD4+ resident memory T cells (TRMs) and HA-specific CD8+ TRMs in lung tissue were quantified by flow cytometry. Data are representative or pooled from two independent experiments. Error bars are shown in mean ± SEM. Statistical significance was tested using one-way ANOVA with Tukey’s multiple comparison test. ns, nonsignificant; *P < 0.05; **P < 0.01; ***P < 0.001; ****P < 0.0001.

Parenteral delivery of mRNA-LNP vaccine induces robust circulating humoral and cellular immune responses (10), but not resident memory CD4+ and CD8+ T cells or secretory IgA in the respiratory tract (11, 12). Given that P+B+HA regimen reduced viral shedding compared to P+B+B, we hypothesized that IN HA boosters elicit robust local immune responses in the respiratory mucosa, the primary site of influenza virus infection and replication. To determine whether the intranasal delivery of recombinant A/PR8 HA protein following two doses of parenteral A/PR8 HA mRNA-LNP vaccination establishes protective mucosal immune memory, we assessed A/PR8 HA-specific humoral and T cell responses 6 wk post–final booster, when memory lymphocyte populations are established. P+B+B led to the induction of systemic antibody responses (Fig. 2 *A*–*C*) and local (Fig. 2*D*) IgG responses but failed to induce local mucosal IgA responses (Fig. 2 *E* and *F*). By contrast, P+B+HA and P+HA+HA resulted in robust mucosal IgA responses in the upper and lower respiratory tracts (Fig. 2 *E* and *F*) in addition to systemic and local IgG responses (Fig. 2 *A*–*D*). We further examined extravascular, tissue-resident memory T cell (TRM) responses in the lung and found a significant increase in polyclonal CD4+ TRMs (as defined by IV-CD69+CD103+CD44+CD4+ T cell, Fig. 2*G* and *SI Appendix*, Fig. S2 *A* and *B*) and antigen-specific CD8+ TRMs (as defined by IV-Tet+CD44+CD8+ T cell, Fig. 2*H* and *SI Appendix*, Fig. S2 *A* and *C*) in the P+B+HA and P+HA+HA groups. Conversely, P+B+B induced extravascular CD8+ TRMs but did not stimulate polyclonal CD4+ TRM responses (Fig. 2 *G* and *H* and *SI Appendix*, Fig. S2 *B* and *C*). These results demonstrated the unique capacity of nasal HA protein boosters to establish robust local mucosal immune memory responses.

### IgG and IgA Are Strong Mucosal Immune Correlates of Protection.

**Fig. 3.**
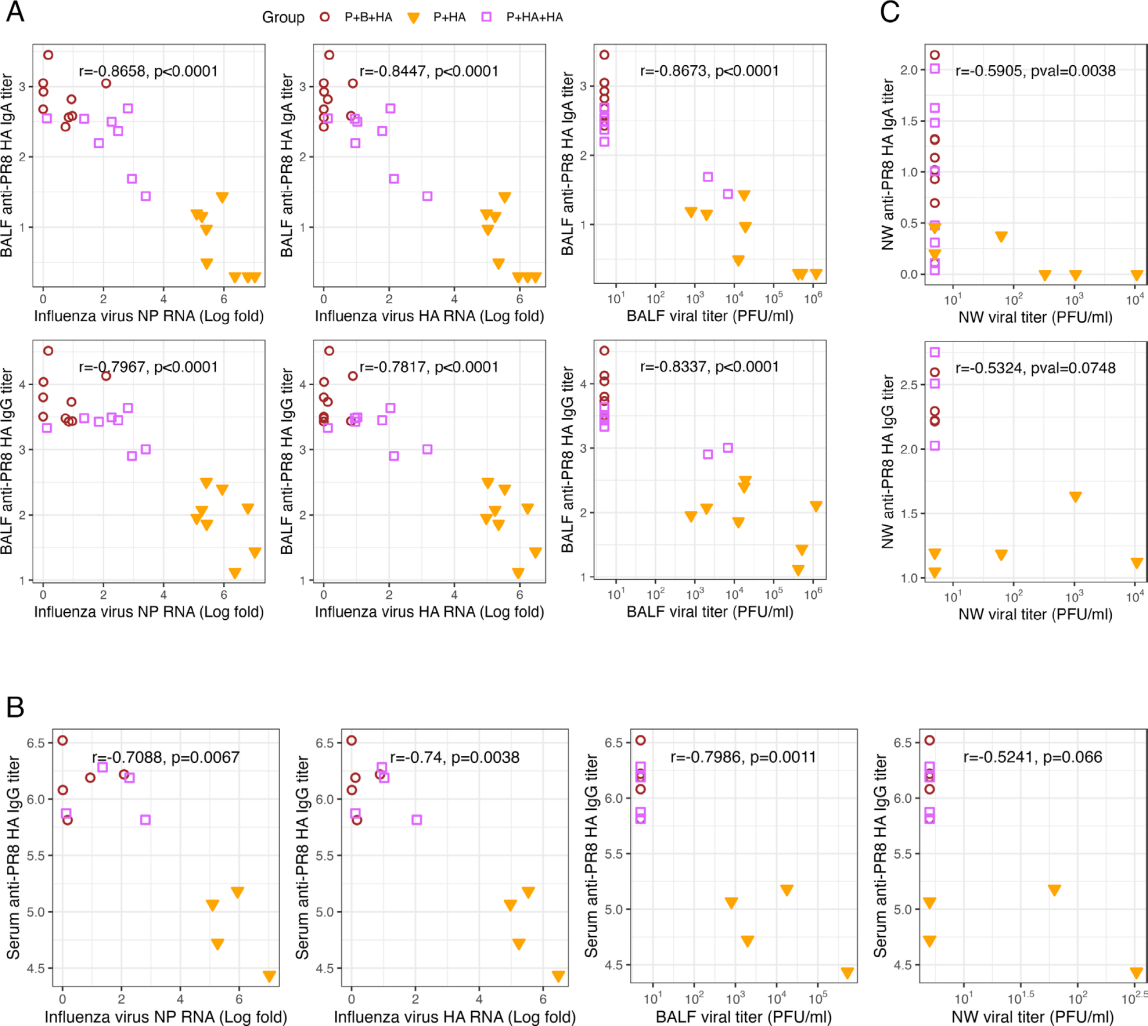
Mucosal IgA and IgG inversely correlate with viral burden. (*A*–*C*) Balb/c mice were immunized with one or two doses of 0.05 μg of PR8 HA mRNA-LNP intramuscularly and boosted with 5 μg recombinant PR8 HA protein intranasally (P+HA or P+B+HA). Another group of Balb/c mice was primed with 0.05 μg of PR8 HA mRNA-LNP intramuscularly and received two doses of 5 μg recombinant PR8 HA booster intranasally (Prime and HA and HA; P+HA+HA). Six weeks after the second boost, mice were challenged with 10^4^ PFU of A/PR8. Nasal wash, BALF, serum, and lung tissues were collected 3 d after infection. Infectious viral titers, viral RNA levels, and PR8 HA-specific antibody responses were determined. Spearman correlations denoted by R between anti-HA antibody titers and either viral titers or viral RNA levels in BALF (*A*), serum (*B*), and nasal wash (*C*). Statistical significance of correlations was assessed using the two-tailed *t* test. *P*-values of <0.05 were considered statistically significant.

Systemic HA-binding antibody titers correlate well with protection to influenza and have long served as a standard for measuring vaccine effectiveness (13). However, to advance the development of next-generation influenza vaccines, a deeper understanding of the correlates of protection, particularly mucosal correlates, is crucial (5, 14, 15). To identify the mucosal correlates of protection against influenza virus infection, we measured A/PR8 HA-specific IgA and IgG levels in the nasal wash and BALF from the same cohort as Fig. 1 *D*–*G*. As collection of nasal wash and BALF requires terminal procedures, the samples are obtained 3 d postinfection, not preinfection. Spearman correlation coefficients were calculated between the levels of mucosal antibodies and protection, as reflected by viral shedding after the challenge. We performed two separate correlation analyses, one with the groups that received IN A/PR8 HA boosters (P+HA, P+B+HA, and P+HA+HA) (Fig. 3) and another with the groups that received various numbers of IM A/PR8 HA mRNA-LNP vaccines (Prime, P+B, and P+B+B) (*SI Appendix*, Fig. S4), as the latter groups lacked mucosal IgA responses (*SI Appendix*, Fig. S3). Our analysis revealed strong and significant negative correlations between BALF A/PR8 HA-specific IgG and both BALF viral titers and lung influenza A/PR8 NP RNA, as well as lung influenza A/PR8 HA RNA regardless of the presence of IgA (Fig. 3 *A*, *Bottom* row and *SI Appendix*, Fig. S4 *A*, *Bottom* row). These negative correlations were as strong as the correlations between serum A/PR8 HA-specific IgG and viral load (Fig. 3*B* and *SI Appendix*, Fig. S4*B*). Further, in the IN-booster cohort, we observed similarly strong negative correlations between BALF A/PR8 HA-specific IgA levels and viral shedding in the BALF (Fig. 3 *A*, *Top* row). Additionally, we found significant negative correlations between nasal wash A/PR8 HA-specific IgA and nasal viral titers (Fig. 3 *C*, *Top* and *SI Appendix*, Fig. S4 *C*, *Top*). The correlation between higher nasal A/PR8 HA-specific IgG titers and lower nasal viral titers was not statistically significant in the IN-booster cohort, likely due to missing data points resulting from limited sample availability (Fig. 3 *C*, *Bottom*). Albeit nonsignificant levels compared to naïve (*SI Appendix*, Fig. S3*A*), nasal wash A/PR8 HA-specific IgA correlated negatively with nasal viral titers in the absence of BALF IgA responses (*SI Appendix*, Fig. S4 *C*, *Top*). These findings demonstrate the negative correlations between the viral load and mucosal IgG and IgA, in addition to serum IgG, in upper and lower respiratory tracts. Furthermore, our analyses reveal distinct correlates of protection in reducing viral load between mucosal and parenteral immunizations. In parenteral regimens, serum IgG showed the strongest negative correlation with viral load, followed by BALF IgG, which largely originates from circulation (16). In contrast, BALF IgA was the most robust correlate of protection after mucosal immunization.

### Intranasal HA Boosters Provide Protective Immune Responses in Older Mice.

**Fig. 4.**
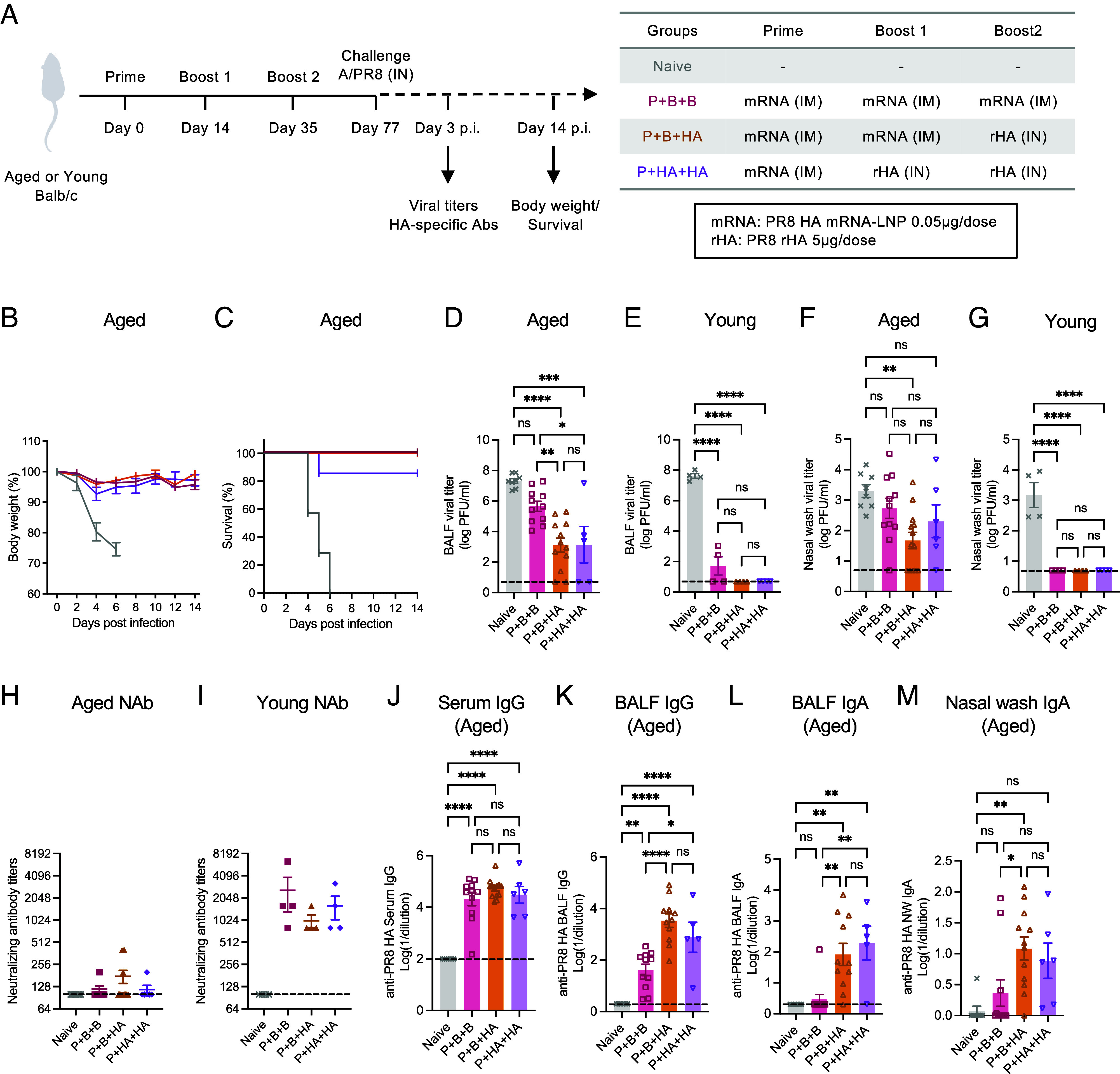
Intranasal HA administration boosts protective immunity in aged mice. (*A*) Aged Balb/c mice were primed and boosted with 0.05 μg of PR8 HA mRNA-LNP intramuscularly and received either an additional dose of PR8 HA mRNA-LNP (Prime and boost and boost; P+B+B) or 5 μg recombinant PR8 HA protein intranasally (Prime and boost and HA; P+B+HA). Another group of aged mice was primed with 0.05 μg of PR8 HA mRNA-LNP intramuscularly and received two doses of 5 μg recombinant PR8 HA booster intranasally (Prime and HA and HA; P+HA+HA). A paired young cohort was prepared for the viral load assessment. Six weeks after the second boost, mice were challenged with 10^4^ PFU of A/PR8. Serum, nasal wash, and BALF were collected 3 d after infection. (*B* and *C*) Body weight and survival were monitored for 14 d after challenge. (*D*–*G*) Viral load in BALF (*D* and *E*) and nasal wash (*F* and *G*) was determined by the plaque assay. (*H* and *I*) Serum neutralizing antibody titers against A/PR8 strain were determined by the microneutralization assay. (*J*–*M*) PR8 HA-specific systemic and mucosal IgG and IgA responses were measured by ELISA. Data are pooled from at least two independent experiments. Error bars are shown in mean ± SEM. Statistical significance was tested using one-way ANOVA with Tukey’s multiple comparison test. ns, nonsignificant; *P < 0.05; **P < 0.01; ***P < 0.001; ****P < 0.0001.

Advanced age is a significant risk factor for influenza virus and other respiratory infections (17). Despite the critical need for protection in this population, the immunogenicity and effectiveness of seasonal influenza vaccines among adults older than 65 y of age are lower than those observed in younger populations (18). To assess whether our vaccination strategy offers enhanced protection in aged hosts, we compared the protective efficacy of the parenteral A/PR8 HA mRNA-LNP vaccination regimen (P+B+B) and intranasal A/PR8 HA boosting regimens (P+B+HA or P+HA+HA) in older mice. Previous studies have shown that both resistance to respiratory viral infection (19) and antibody responses following initial series of mRNA-LNP immunization (20) are reduced in 1-y-old mice in the former study and 11-mo-old mice in the latter, compared to younger mice. Female Balb/c mice aged 14 to 20 mo (age-matched mice were evenly distributed to each group per each experiment) were immunized with P+B+B, P+B+HA, or P+HA+HA regimens, and their protective efficacy was evaluated after 6 wk by monitoring body weight, survival, and viral burden after challenge with A/PR8 virus (Fig. 4*A*). All vaccinated mice, except for one out of seven immunized with P+HA+HA, survived and were protected from weight loss following an otherwise 100% lethal infection (Fig. 4 *B* and *C*). Although P+B+HA significantly reduced viral shedding in both upper and lower respiratory tracts, a considerable viral burden remained (Fig. 4 *D* and *F* and *SI Appendix*, Fig. S5) in older mice. Yet, intranasally boosted groups showed significantly reduced lung viral loads by 10,000-fold compared to naïve, offering more efficient protection than P+B+B (Fig. 4*D* and *SI Appendix*, Fig. S5). In contrast, the same immunization regimens provided a substantial reduction in viral burden in younger mice (Fig. 4 *E* and *G* and *SI Appendix*, Fig. S5), illustrating the attenuated vaccine-induced protection in aged hosts. Consistent with the striking age-associated difference in vaccine-induced protection, high serum neutralizing antibody titers were seen in younger mice while the titers in older mice were close to the limit of detection (Fig. 4 *H* and *I*). Since we observed similar levels of serum neutralizing antibodies and HA-specific IgG among different vaccine groups (Fig. 4 *H* and *J*), we hypothesized that the superior reduction in respiratory viral load observed with intranasal boosting was associated with stronger mucosal antibody responses. Indeed, P+B+HA elicited higher mucosal HA-specific IgG and IgA responses compared with P+B+B (Fig. 4 *K*–*M*). Notably, P+HA+HA also induced strong BALF HA-specific antibodies comparable to P+B+HA (Fig. 4 *K* and *L*). These data indicate that the mucosal HA booster provides better mucosal immune responses than IM mRNA-LNP booster, correlating with a significantly lower viral replication in aged mice.

### Intranasal Heterosubtypic H5 HA Boosters Generate H5-Reactive Humoral Immunity in H1N1 Preexposed Mice.

**Fig. 5.**
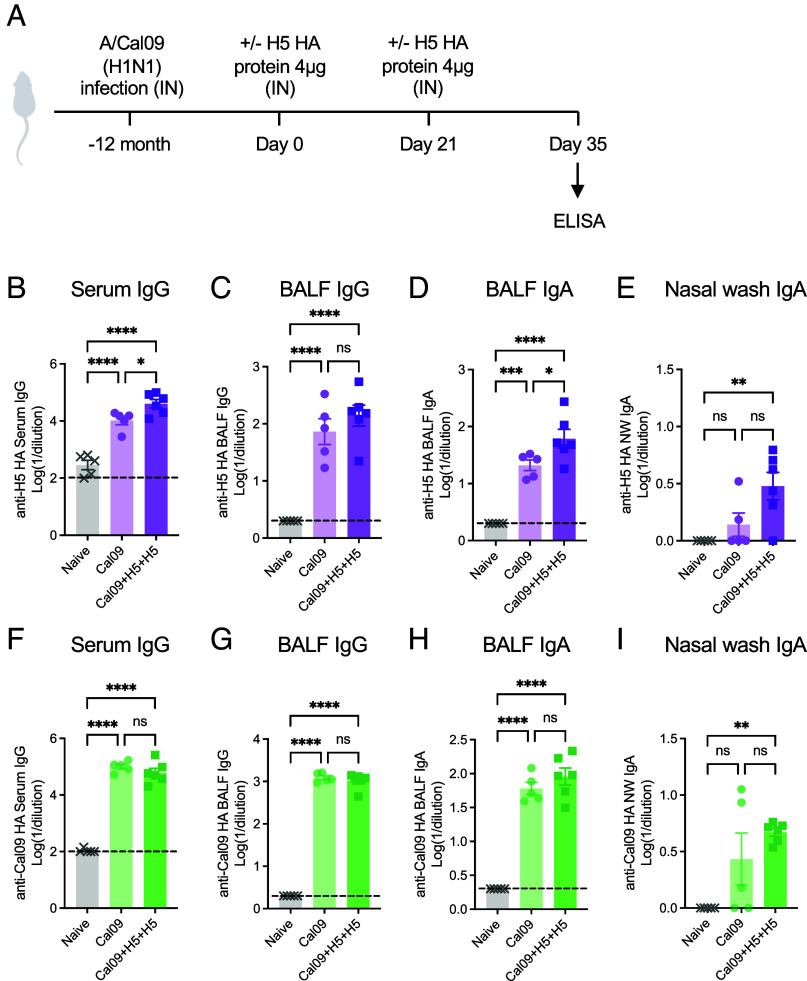
Intranasal heterosubtypic H5 HA boosters elicit H5-reacting antibody responses. (*A*) Balb/c mice infected with a sublethal dose (10^5^ PFU) of A/California/04/09 (H1N1) 12 mo prior were immunized twice with 4 μg recombinant H5 (VN04; A/Vietnam/1194/2004) HA protein intranasally (Cal09+H5+H5) or remained untreated (Cal09). Two weeks after the second boost, serum, nasal wash, and BALF were collected and analyzed for VN04-reacting antibody responses. (*B*–*I*) VN04 HA-specific (*B*–*E*) or Cal09 HA-specific (*F*–*I*) systemic and mucosal IgG and IgA responses were measured by ELISA. Data are representative of two similar experiments. Error bars are shown in mean ± SEM. Statistical significance was tested using one-way ANOVA with Tukey’s multiple comparison test. ns, nonsignificant; *P < 0.05; **P < 0.01; ***P < 0.001.

Next, we investigated whether the intranasal boosting with H5 HA protein could induce H5-reactive mucosal humoral responses in the hosts previously exposed to an H1N1 viral infection. Balb/c mice which were infected with a sublethal dose of A/California/04/09 (Cal09) strain 12 mo prior were intranasally boosted with 4 μg of H5 HA protein from A/Vietnam/1194/2004 strain (H5N1; VN04) twice (Fig. 5*A*). The prior infection with Cal09 alone resulted in significant cross-reactive antibody responses against VN04 H5 HA, except for VN04 H5-specific IgA in the nasal wash (Fig. 5 *B*–*E*). After two doses of intranasal VN04 H5 booster, there was a marked elevation in systemic (Fig. 5*B*), but not local (Fig. 5*C*) VN04 H5-specific IgG responses. Mucosal VN04 H5-specific IgA responses were significantly elevated by intranasal VN04 H5 booster over prior infection with Cal09 alone in BALF (Fig. 5*D*), and significantly above naïve mice in the nasal wash (Fig. 5*E*). In contrast, antibodies specific to the initially exposed Cal09 H1 remained largely unchanged (Fig. 5 *F*–*I*). These findings demonstrate the ability of intranasal heterosubtypic H5 HA protein boosters to enhance H5-reactive humoral immune responses, extending beyond the influenza virus subtype previously encountered.

### Intranasal Bivalent H1 HA Boosters Elicit Robust Immunity to Both PR8 and MI15.

**Fig. 6.**
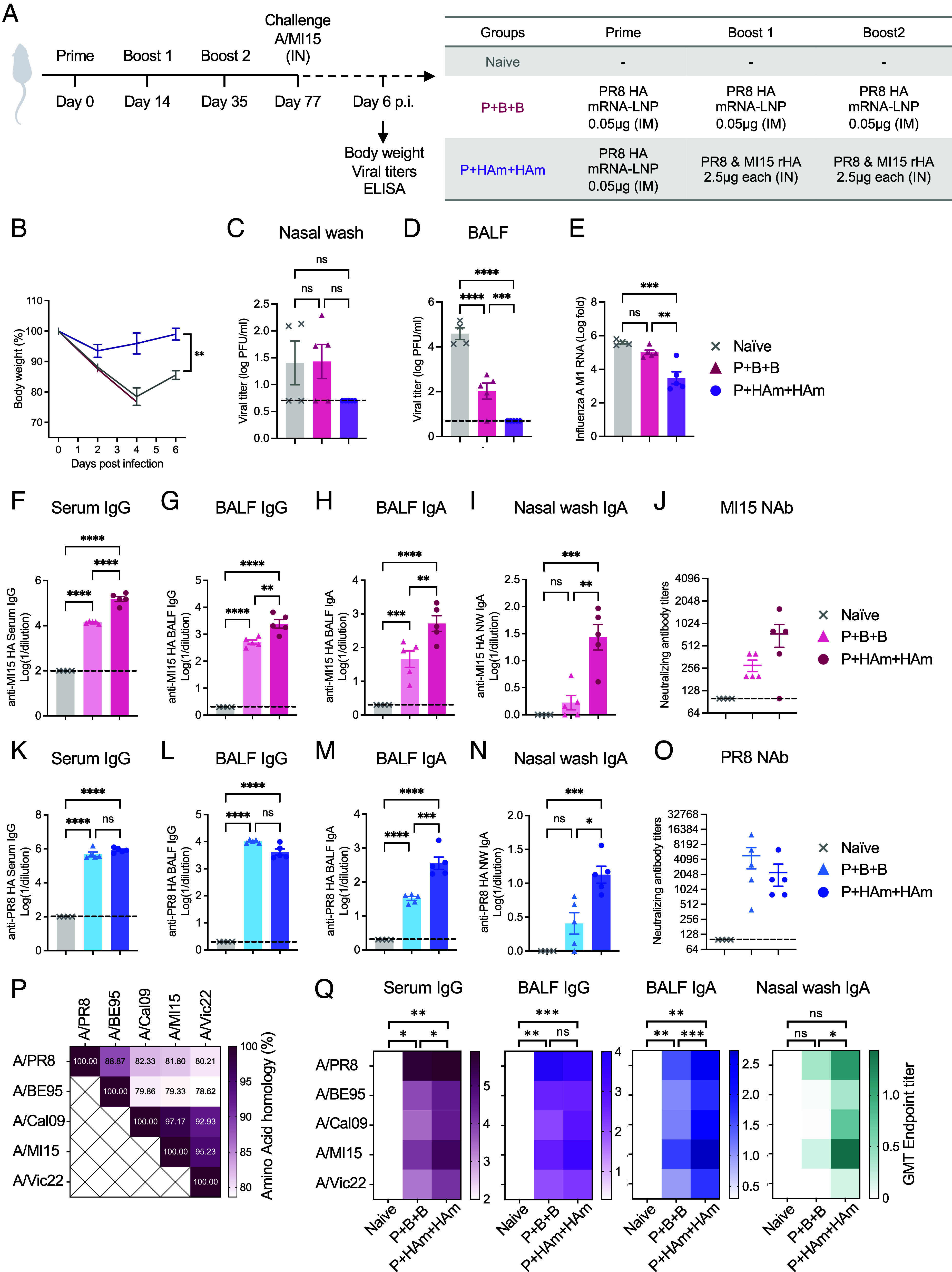
Bivalent intranasal HA boosters elicit heterologous mucosal antibody responses in the presence of A/PR8-specific preexisting immunity. (*A*) Balb/c mice were primed with 0.05 μg of PR8 HA mRNA-LNP intramuscularly and boosted twice with recombinant HA cocktail (2.5 μg each of PR8 and MI15 HA) intranasally (Prime and HA mix and HA mix; P+HAm+HAm) or received three doses of PR8 HA mRNA-LNP intramuscularly (Prime and boost and boost; P+B+B). Six weeks after the second boost, mice were challenged with 5,000 PFU of A/Michigan/45/2015. Body weight was monitored for 6 d (*B*). On day 6, serum, nasal wash, and BALF were collected. (*C* and *D*) Viral load in nasal wash (*C*) and BALF (*D*) was determined by the plaque assay. Viral RNA levels in the lung tissue were determined by RT-qPCR using primers targeting Influenza A M1 gene (*E*). (*F*–*O*) MI15 HA-specific (*F–I*) or PR8 HA-specific (*K*–*N*) systemic and mucosal IgG and IgA responses were measured by ELISA. (*J* and *O*) Serum neutralizing antibody titers against MI15 strain (*J*) and PR8 strain (*O*) were determined by the microneutralization assay. (*P*) Amino acid sequence homology between the H1 HA used for ELISA is shown. A/PR8: A/Puerto Rico/8/1934, A/BE95: A/Beijing/262/1995, A/Cal09: A/California/04/2009, A/MI15: A/Michigan/45/2015, A/Vic22: A/Victoria/4897/2022. (*Q*) H1 HA-binding systemic and mucosal antibodies were measured by ELISA. Data are representative of two similar independent experiments. Error bars are shown in mean ± SEM. Statistical significance was tested using two-way ANOVA for (*B*) and one-way ANOVA with Tukey’s multiple comparison test for the rest of the figures. ns, nonsignificant; *P < 0.05; **P < 0.01; ***P < 0.001; ****P < 0.0001.

The antigenic evolution of seasonal influenza viruses substantially decreases the efficacy of seasonal influenza vaccines from 1 y to the next (14). We sought to assess the potential of intranasal booster with A/Michigan/45/2015 (H1N1; MI15) HA booster to elicit antibody reactivity against MI15 strain in animals previously immunized against A/PR8 HA. As we observed the increased antibody responses against the priming strain (A/PR8) in addition to the boosting strain (Cal09) by administering multiple strains of HA antigens than with single heterologous H1 HA antigen (*SI Appendix*, Fig. S6), we decided to proceed with a bivalent intranasal HA booster. Mice were primed with mRNA-LNP vaccine encoding PR8 HA and then intranasally boosted twice with a bivalent HA cocktail containing 2.5 μg each of PR8 HA and MI15 HA protein, which differ in HA amino acid sequence by 18.2% (Prime and HA mix and HA mix; P+HAm+HAm). As a control, another group received three doses of PR8 HA mRNA-LNP (P+B+B) (Fig. 6*A*). Six weeks after the final dose, the immunized mice were challenged with an MI15 strain and assessed for protection based on weight loss, viral load, and MI15 HA-binding antibody responses. The mice immunized with P+HAm+HAm exhibited only slight weight loss on day 2 postinfection but quickly recovered (Fig. 6*B*). In addition, there was no detectable infectious viral load in either the nasal wash (Fig. 6*C*) or the BALF (Fig. 6*D*) and reduced viral RNA in the lung (Fig. 6*E*) of P+HAm+HAm mice, indicating that the intranasal bivalent HA boosters elicited robust protective immunity against MI15 HA. Consistently, significant induction of systemic and mucosal MI15 HA-specific IgG (Fig. 6 *F* and *G*) and mucosal IgA (Fig. 6 *H* and *I*), as well as serum MI15-neutralizing antibodies (Fig. 6*J*) was observed in the P+HAm+HAm group. By contrast, mice immunized with P+B+B lost body weight to levels similar to the Naïve group (Fig. 6*B*). P+B+B did not alter the viral titers in the nasal wash (Fig. 6*C*) but reduced titers in the BALF by more than 100-fold compared to the naive group (Fig. 6*D*). This reduction in BALF viral titer was associated with the elevation of MI15 HA-reactive IgG and IgA responses in the serum and BALF (Fig. 6 *F*–*H*). The induction of BALF IgA contrasts with previous results, where BALF IgA responses were absent in P+B+B-vaccinated mice without viral challenge (Fig. 2*E*) or at 3 d post-A/PR8 challenge (Fig. 4*L* and *SI Appendix*, Fig. S3*B*). Since the sample collection here was performed at day 6 postinfection, the BALF IgA detected is more likely attributable to responses against the MI15 challenge, rather than sorely to parenteral vaccination. In addition to the robust antibody responses against MI15, bivalent intranasal HA booster induced IgG responses (Fig. 6 *K* and *L*), mucosal IgA responses (Fig. 6 *M* and *N*), and neutralizing antibodies (Fig. 6*O*) against A/PR8 to the similar or higher levels as A/PR8 HA mRNA-LNP booster. We further assessed antibody responses against vaccine-mismatched H1 HAs (Fig. 6*P*) and observed enhanced systemic and mucosal antibody responses beyond vaccinated strains with P+HAm+HAm (Fig. 6*Q* and *SI Appendix*, Fig. S7). Collectively, these data demonstrate that intranasal bivalent H1 HA booster containing A/PR8 and MI15 HA confers robust protection against the MI15 strain while enhancing cross-reactive antibodies to heterologous H1.

## Discussion

In this study, we demonstrate the ability of IN HA protein boosters following IM HA-encoding mRNA-LNP priming to elicit robust protective mucosal immune responses and confer clinical and virological protection against influenza virus infection. While IN HA booster and IM mRNA-LNP booster similarly induced systemic and local HA-specific IgG responses, mucosal anti-HA IgA antibodies were uniquely induced by the IN HA booster. Protection conferred by Prime and HA lasts at least for 12 wk and was demonstrated even in older mice. By using bivalent H1 HA boosters, we were able to demonstrate the enhanced mucosal antibody responses against both vaccine-matched and -unmatched heterologous H1 HA. The simplicity of needle-free booster administration emphasizes the potential of IN HA booster as an alternative strategy to reduce disease severity. Our findings also validate that the previously established “Prime and Spike” approach (7) can be effectively applied to different pathogens. Crucially, Prime and HA provides an unadjuvanted mucosal vaccine approach to establish local protection against infection, thus inhibiting replication of influenza virus.

Here, we used the mRNA-LNP platform or previous virus infection to generate preexisting systemic immunity, which allowed an intranasal adjuvant-free recombinant HA protein booster to induce potent respiratory mucosal immune responses. Even though an IN HA booster moderately promoted the recovery of QIV-primed mice, its booster effect was significantly more pronounced in mRNA-LNP-primed mice. Previous research has shown that a single intradermal mRNA-LNP injection generates robust splenic single and polyfunctional CD4+ and follicular helper T cells (TFH) in mice, unlike IM immunization with inactivated influenza virus (21–23). The variability in the booster effect likely reflects differences in the quality and magnitude of immune responses induced by different priming vaccines.

We demonstrated that IM prime-boost immunization of HA encoding mRNA-LNP followed by an IN recombinant HA booster, which we term P+B+HA, elicits robust mucosal immune responses. The P+B+HA regimen not only prevented disease progression and lung pathology but also provided robust virological protection against a lethal influenza virus challenge, a protection not afforded by parenteral mRNA-LNP vaccination control (P+B+B). Similarly, single IM HA mRNA-LNP immunization followed by two doses of IN recombinant HA booster, termed P+HA+HA, also elicited mucosal immune responses and was superior to P+B+B at reducing viral load in the lower respiratory tract in aged mice. The enhanced protection with P+B+HA and P+HA+HA was accompanied by robust mucosal IgA responses in upper and lower respiratory tracts, suggesting the substantial role of mucosal IgA antibodies in limiting viral replication. The importance of local IgA antibodies is further supported by their significant negative Spearman correlations with viral shedding in BALF (Fig. 3*A*) and the inverse relationship between nasal IgA levels and nasal viral titer (Fig. 3*C*). These results align with previous studies illustrating the importance of nasal secretory IgA in reducing viral shedding in humans (24, 25) and underscore the importance of local mucosal immunity as the first line of defense in the respiratory mucosa. Recent studies on COVID-19 vaccines have shown that mucosal vaccines delivered via inhalation, intranasal, or intratracheal routes can induce robust protective immunity in upper and lower respiratory tracts in nonhuman primates and humans (8, 26, 27). For instance, Ye et al. and Darling et al. reported that their nanoparticle-based inhaled vaccine or chimpanzee adenoviral-vectored intranasal vaccine prevents aerosol transmission of SARS-CoV-2 in hamsters (27, 28). Future research should explore whether our nasal booster approach can efficiently prevent viral transmission.

Developing alternative vaccine strategies to improve protection for the older adults who are at higher risk of developing severe disease is a key focus for next-generation influenza vaccines. Successful vaccination of older adults is a benchmark for novel vaccine approaches, given their typically reduced responsiveness compared to younger populations (15). To enhance the immunogenicity of seasonal influenza vaccines in older adults, MF-59 adjuvanted or high-dose formulations have been approved (29, 30). In this study, we employed the potent mRNA-LNP platform for priming and assessed the protective efficacy of IN HA booster versus standard IM mRNA-LNP booster in aged mice. Consistent with findings in young mice, P+B+HA and P+HA+HA mounted higher local antibody responses and significantly reduced viral burden compared with the standard P+B+B control in older hosts with weakened immune systems. Given the engagement of distinct immune cell populations and signaling pathways, the boosting effect of IN HA and IM mRNA-LNP can be differently impacted by aging. While mRNA-LNP vaccines elicit multitude of innate sensors for adaptive immune activation (31–33), aging-related degradation of TRAF3 and diminished IFN expression may hinder the adjuvanticity of the vaccines (34). Nevertheless, for transmission-blocking vaccine strategy, our data show that IN HA protein booster is still effective in reducing viral load in older mice.

Limitations of our study include the use of inbred mice, which may not reflect the immunology or physiology of human respiratory tract. Second is the lack of demonstration of transmission-blocking capabilities of the Prime and HA approach. Since mice are not naturally capable of transmission (35), future studies involving established models of transmission such as ferrets are needed to address this question. Considering that the high dose formulations are used for seasonal influenza vaccinations in older adults, determining optimal intranasal booster dosing for young and elderly cohort would be important. This includes the dose escalation studies while considering the human-equivalent dose per weight. In parallel, optimizing mucosal delivery of HA antigen, such as the use of clinically approved adhesive excipients that prolong antigen retention (36) and enhance mucosal immune responses (37), may allow for reduced antigen input and is an important area of exploration. The use of different ages of mice, immunization and challenge schedules, immune status at the time of immunization, virus strains, and vaccine formulations may result in variations of immunogenicity and challenge outcomes across experiments.

One of the major challenges for influenza vaccines is the emergence of immune escape mutants due to the rapid antigenic evolution of the viral surface glycoproteins. In this study, we addressed this challenge by demonstrating two sequential doses of IN heterologous or heterosubtypic HA boosters to elicit antibody responses against the booster-matched HA in mice previously vaccinated or infected. Administering a bivalent HA booster containing the primary vaccine-matched A/PR8 HA and antigenically diverged MI15 H1 HA protein as an IN booster elicited protective mucosal immunity against MI15 HA and antibodies targeting heterologous H1 HA. Given the potential pandemic threat posed by the recently emerged bovine H5N1 influenza (38), we also explored the potential of IN H5 HA booster to establish H5-reactive immunity in hosts with prior exposure to an H1N1 virus. Heterosubtypic H5 HA IN booster doses were able to boost H5-reactive mucosal IgA responses in mice that recovered from an H1N1 virus 1 y prior. As antibody titers against the original H1 antigen remained unchanged after H5 HA boosting (Fig. 5 *F*–*I*), the observed increase in H5-specific antibodies is likely attributable to a de novo response to the H5 antigen. This interpretation, however, requires further validation through approaches such as preclearing the samples with the original H1 antigen and molecular approaches of longitudinal BCR sequencing. The promise of the “Prime and HA” to confer protective immunity in humans merits further investigation.

## Materials and Methods

### Study Design.

The aim of this study was to evaluate the efficacy of an intranasal unadjuvanted HA protein booster following intramuscular mRNA-LNP priming. This included a comprehensive virological and immunological assessment, such as the detection of HA-specific resident memory T cells using H-2K(d) tetramers. We employed a well-established mouse model of influenza virus infection, utilizing Balb/c mice and the mouse-adapted A/Puerto Rico/8/34 (H1N1) strain. A/Michigan/45/2015 (H1N1) strain was used to evaluate protection in mice receiving a bivalent HA booster.

### Mice.

Six- to 10-wk-old female Balb/c mice were purchased from Charles River Laboratories or bred in-house. Fourteen- to 20-mo-old female Balb/c mice were used as aged mice. All animal experiments in this study complied with federal and institutional policies of the Yale Animal Care and Use Committee.

### Cells and Viruses.

Madin-Darby canine kidney (MDCK) cells were maintained in MEM supplemented with 1% Penicillin-Streptomycin and 10% heat-inactivated fetal bovine serum (FBS) at 37 °C. A/Puerto Rico/8/34 (H1N1) was kindly provided by Hideki Hasegawa (National Institute of Infectious Diseases, Japan). A/California/04/09 (H1N1) was kindly provided by Adolfo Garcia-Sastre (Icahn School of Medicine at Mount Sinai). A/Michigan/45/2015 (H1N1) was kindly provided by Michael Schotsaert (Icahn School of Medicine at Mount Sinai). Virus stocks were propagated in allantoic cavities from 10- to 11-d-old fertile chicken eggs for 2 d at 35 °C. Viral titers were determined by the standard plaque assay procedure. Briefly, serial 10-fold dilutions of nasal wash and BALF were prepared in PBS containing 0.1% bovine serum albumin (BSA). Aliquots of 200 μL of diluted samples were inoculated into a confluent monolayer of MDCK cells in 6-well plates and incubated for 1 h at 37 °C. After incubation, cells were washed with PBS and overlaid with 2 mL agar-containing MEM supplemented with TPCK-treated trypsin. Forty-eight hours later, cells were stained with crystal violet, and the number of plaques in each well was counted. All experiments using live influenza virus were performed in biosafety level 2 laboratories and animal facilities with approval from the Yale Environmental Health and Safety office.

### Vaccines and Proteins.

Nucleoside-modified, A/Puerto Rico/8/1934 or A/Michigan/45/2015 HA-encoding mRNA-LNPs were kindly provided by Drew Weissman (University of Pennsylvania). Fluzone quadrivalent influenza vaccine 2017-2018 (QIV) was obtained from BEI resources. Recombinant A/Puerto Rico/8/34 (H1N1) HA (Cat#11684-V08H1), A/Beijing/262/1995 (H1N1) HA (Cat#40133-V08B-100), A/California/04/09 (H1N1) HA (Cat#11055-V08H), A/Michigan/45/2015 (H1N1) HA (Cat#40567-V08H1), and A/Vietnam/1194/2004 (H5N1) (Cat#11062-V08H1) were purchased from Sino Biological. Recombinant A/Victoria/4897/2022 (H1N1) HA was kindly provided by Florian Krammer (Icahn School of Medicine at Mount Sinai).

### Vaccination and Infection.

For intramuscular immunization, mice were anesthetized by isoflurane inhalation and injected with 0.05 μg mRNA-LNP or 25 μL QIV (containing 0.75 μg H1 HA) adjusted to 50 μL in PBS. For intranasal vaccination or infection, mice were fully anesthetized by intraperitoneal injection of ketamine and xylazine, and intranasally inoculated with 50 μL of PBS containing recombinant HA protein or influenza virus.

### Microneutralization Assay.

Neutralizing antibody titers against influenza A viruses were determined by the microneutralization assay as previously described with some modifications (39). Briefly, serum samples were treated with receptor-destroying enzyme (RDE) for 20 h at 37 °C, followed by heat inactivation at 56 °C for 30 min. Serial twofold dilutions of inactivated sera were prepared in 96-well U-bottom plates in virus diluent (DMEM containing 21.7 mM HEPES, 1% bovine albumin fraction V, and Penicillin-Streptomycin) supplemented with TPCK-treated trypsin. An equal volume of 100 TCID A/PR8 or A/MI15 virus-containing virus diluent was added to each well and incubated for 1 h at 37 °C. After incubation, a confluent monolayer of MDCK cells in 96-well plates was washed with PBS twice and overlaid with 100 μL of serum-virus mixture. After 3 d incubation, cells were stained with crystal violet, and the neutralizing antibody titers were determined as reciprocal of the highest dilution that completely inhibits cell death.

### Measurement of Anti-HA Antibodies.

Influenza HA-reacting antibodies were detected by the enzyme-linked immunosorbent assay (ELISA) as previously described (16). Briefly, 96-well or 384-well MaxiSorp plates were coated with 2 μg/mL of HA protein in carbonate buffer overnight at 4 °C and blocked with PBS containing 4% FBS for 1 h at room temperature. Serum, nasal wash, and BALF samples were diluted in PBS containing 4% FBS and applied to the plates. Following overnight incubation at 4 °C, plates were washed with 0.1% Tween-20 containing PBS and incubated with horseradish peroxidase-conjugated anti-mouse IgG or IgA. After 1 h of incubation at room temperature, plates were washed with 0.1% Tween-20 containing PBS. TMB substrate (eBioscience) was added to each well, and 1N sulfuric acid was added to terminate the reaction. The absorbance of 450 nm wavelength was recorded. Endpoint titers were calculated using curve fitting analysis, with the cut-off defined as the mean plus two SD of the naïve control signals.

### Intravenous Labeling and Lung Cell Isolation.

Circulating lymphocytes were labeled by the previously described intravenous labeling method (7). Briefly, the APC-Cy7-labeled anti-mouse CD45 antibody (Cat#103116, BioLegend) was diluted in PBS and injected intravenously 3 min before the tissue harvest. Lungs were harvested and processed as previously described (11). Briefly, lungs were minced with scissors and digested in RPMI1640 media containing 1 mg/mL collagenase A, 30 μg/mL DNase I at 37 °C for 45 min. Digested lungs were then filtered through a 70 μm cell strainer and treated with ACK buffer for 2 min. After washing with PBS, cells were resuspended in PBS with 1% FBS and subjected to surface marker staining.

### Flow Cytometry.

Cells were blocked with mouse Fc Block in the presence of Live/Dead Fixable Aqua (Cat# L34966, 1:1,000, Thermo Fisher) for 20 min at 4 °C. Staining antibodies were added and incubated for 30 min at 4 °C. The stained cells were washed with 2 mM EDTA-PBS and resuspended in 100 μL 2% PFA for 15 min at 4 °C. After fixation, cells were washed and resuspended in PBS with 1% FBS and analyzed on Attune NxT (Thermo Fisher). The data obtained were analyzed using FlowJo software (Tree Star). Staining antibodies are as follows [PerCP-Cy5.5 anti-mouse CD8α (Cat#103057, 1:400, BioLegend), PE-Cy7 anti-mouse CD69 (Cat#104512, 1:200, BioLegend), AF700 anti-mouse CD4 (Cat#100536, 1:200, BioLegend), BV421 anti-mouse CD103 (Cat#121422, 1:200, BioLegend), BV605 anti-mouse TCRβ (Cat# 109241, 1:200, BioLegend), BV711 anti-mouse CD44 (Cat# 103057, 1:200, BioLegend)]. PE-conjugated H-2K(d) tetramer (Influenza A HA 533-541 IYSTVASSL) was provided by the NIH Tetramer Core Facility.

### Quantitative PCR.

Mouse lung tissues were minced with scissors and lysed with TRIzol reagent (Invitrogen). Total RNA was extracted using an RNeasy mini kit (QIAGEN) and reverse transcribed into complementary DNA (cDNA) using an iScript cDNA synthesis kit (Bio-Rad). RT-PCR was performed by CFX96 Touch Real-Time PCR detection system (Bio-Rad) using iTaq SYBR premix (Bio-Rad) and the following primers (5’-3’): PR8 NP (Forward: AGAACATCTGACATGAGGAC, Reverse: GTCAAAGGAAGGCACGATC), PR8 HA (Forward: AGTGCCCAAAATACGTCAGG, Reverse: GGCAATGGCTCCAAATAGAC), and Influenza A M1 (Forward: GACCRATCCTGTCACCTCTGAC, Reverse: AGGGCATTYTGGACAAAKCGTCTA).

### Pathological Assessment.

Lung tissue was fixed in 4% paraformaldehyde overnight and transferred into 70% ethanol. Paraffin embedding, sectioning, and hematoxylin and eosin (H&E) staining of the tissue were performed by Yale Pathology Tissue Services. Pathological findings were identified and scored by a pulmonary pathologist through blinded sample evaluation using the following criteria: Airway injury is identified based on a composite of the evaluation of morphologic cell death and degeneration and inflammation including acute inflammation using the percent and extent of the circumference involved. Immune cell infiltration is determined based on 1, minimum visible infiltration; and 4, circumferential massive infiltration of lymphocytes and eosinophils. Notably, in all cases, the extent of inflammation was relatively low, and the score was primarily driven by epithelial injury.

### Statistical Analysis.

Statistical significance was tested using one-way ANOVA with Tukey’s multiple comparison test unless otherwise stated. A two-way ANOVA test or a two-tailed *t* test was used where indicated. For correlation analysis, Spearman rank correlation coefficients were calculated to capture all monotonic relationships. No missing data were present in the cohort that received IN HA boosters, and no obvious outliers were observed upon visual inspection of the data distribution. Statistical significance of correlations was assessed using the two-tailed *t* test. *P*-values of <0.05 were considered statistically significant. Correlation analyses were performed using R (version 4.4.1). Prism 9 was used for all other statistical analyses.

## Supplementary Material

Appendix 01 (PDF)

Code S01 (R)

Code S02 (R)

## Data Availability

All study data and R code are included in the article and/or *SI Appendix*.
