# Supplementary material for: Intranasal hemagglutinin protein boosters induce protective mucosal immunity against influenza A viruses in mice
Source: Proc Natl Acad Sci U S A. 2025 Sep 24;122(39):e2422171122. doi: 10.1073/pnas.2422171122 (PMC12501169; doi:10.1073/pnas.2422171122)
Supplement: Supplementary file 1 — Appendix 01 (PDF) [file pnas.2422171122.sapp.pdf]

**Supporting Information for**

# Intranasal hemagglutinin protein boosters induce protective mucosal immunity against influenza A viruses in mice

Miyu Moriyama, Gisele Rodrigues, Jiping Wang, Radeesha Jayewickreme, Andrew Hudak, Huiping Dong, Robert J. Homer, Shuangge Ma, Akiko Iwasaki*

*Address correspondence to Akiko Iwasaki.
Email: akiko.iwasaki@yale.edu

**This PDF file includes:**

Figures S1 to S7

## Supplementary Figures

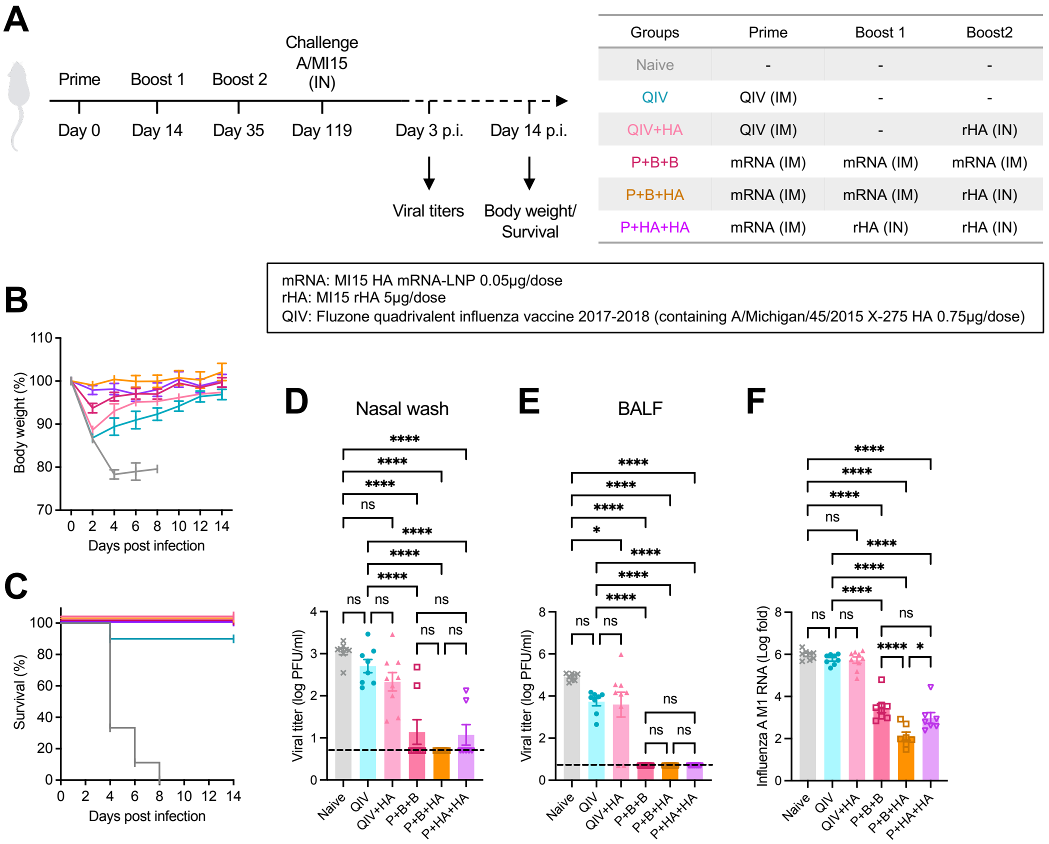


**Fig. S1. Protective efficacy of intranasal HA boosters sustains for at least 12 weeks**
(A) Balb/c mice were immunized with 0.05µg of A/Michigan/45/2015 (MI15) HA mRNA-LNP intramuscularly and 5µg recombinant MI15 HA protein intranasally as indicated. Quadrivalent influenza vaccine (QIV) served as a standard of care control. Twelve weeks after the second boost, mice were challenged with $10^4$ PFU of A/Michigan/45/2015. (B and C) Body weight and survival were monitored for 14 days after the challenge. (D to F) Nasal wash, BALF, and lung tissue were collected 3 days after infection, and infectious viral titers in the nasal wash (D) and BALF (E) were determined by plaque assay. Viral RNA levels in the lung tissue were determined by RT-qPCR using primers targeting Influenza A M1 gene (F). Data are pooled from two independent experiments. Error bars are shown in mean+/- S.E.M. Statistical significance was tested using one-way analysis of variance (ANOVA) with Tukey's multiple comparison test. ns, non-significant; *, p<0.05; ****, p< 0.0001

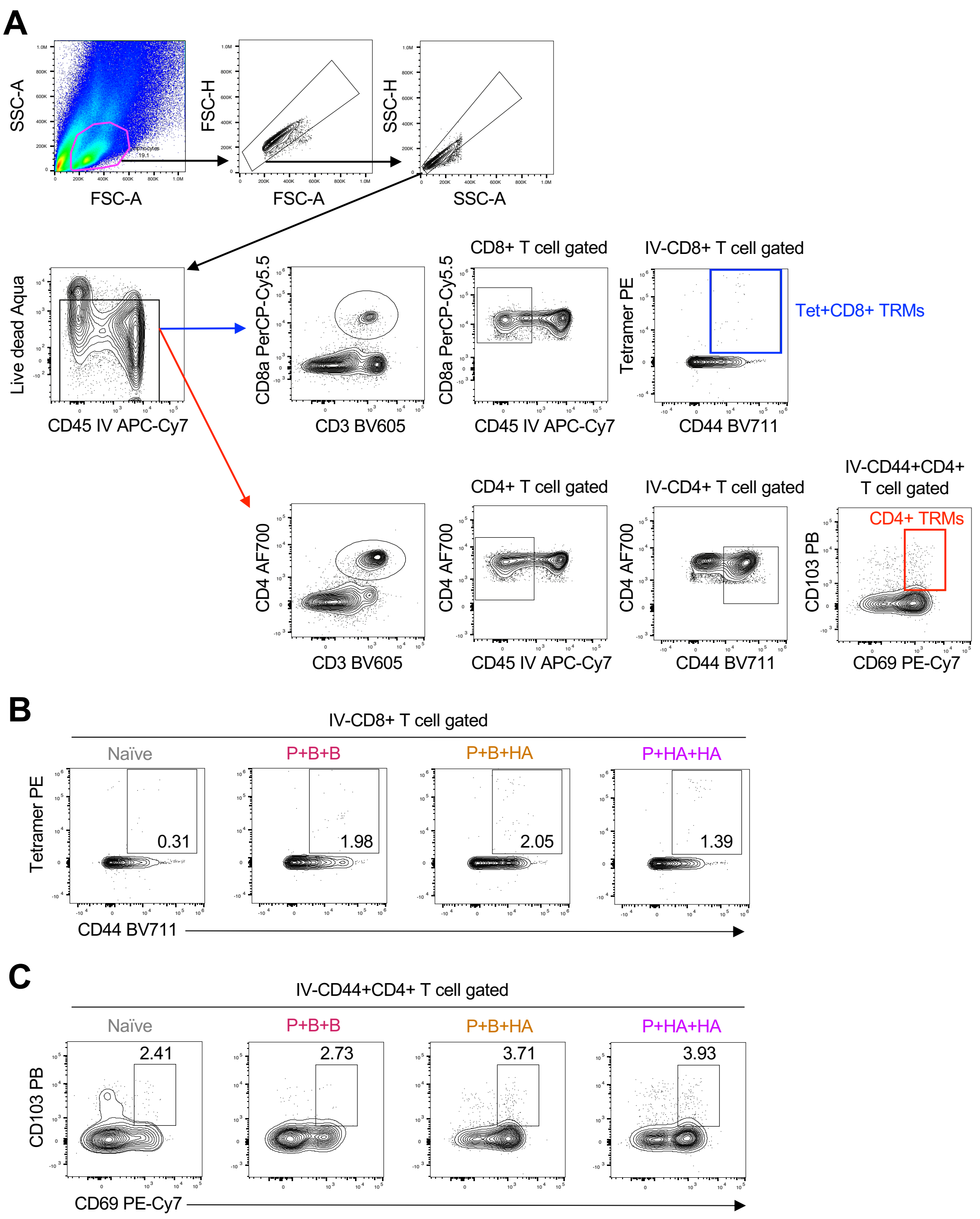


**Fig. S2. Gating strategy and representative flow cytometry plots**
(A to C) Balb/c mice were primed and boosted with 0.05µg of PR8 HA mRNA-LNP intramuscularly and received either an additional dose of PR8 HA mRNA-LNP (Prime and boost and boost; P+B+B) or 5µg recombinant PR8 HA protein intranasally (Prime and boost and HA; P+B+HA). Another group of Balb/c mice were primed with 0.05µg of PR8 HA mRNA-LNP intramuscularly and received two doses of 5µg recombinant PR8 HA booster intranasally (Prime and HA and HA; P+HA+HA). Six weeks after the second boost, lung tissues were corrected and assessed for cellular immune responses. (A) Gating strategy for IV-Tet+CD44+CD8+ T cells (middle row, blue square) and IV-CD69+CD103+CD44+CD4+ T cells (bottom row, red square). (B) Representative flow cytometry plots of IV-Tet+CD44+CD8+ T cells. (C) Representative flow cytometry plots of IV-CD69+CD103+CD44+CD4+ T cells.

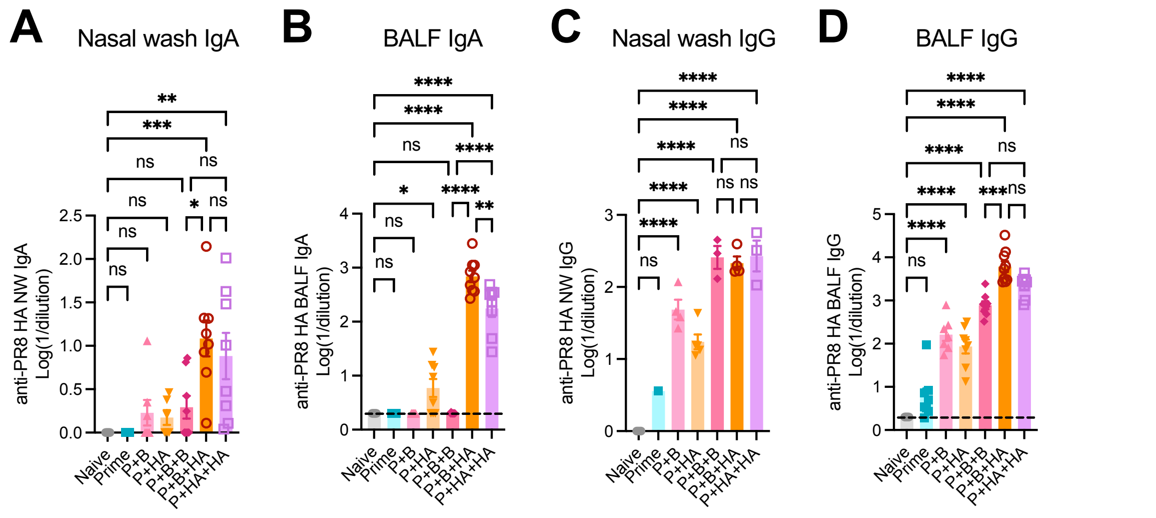


**Fig. S3. HA-specific humoral immune responses in various vaccination regimens**
(A to D) Balb/c mice were immunized with 0.05µg of PR8 HA mRNA-LNP intramuscularly and 5µg recombinant PR8 HA protein intranasally as indicated in Figure 1A. Six weeks after the second boost, mice were challenged with $10^4$ PFU of A/PR8. Three days after infection, nasal wash and BALF were collected and subjected to the measurement of PR8 HA-specific IgG and IgA responses by ELISA. Data are pooled from two independent experiments. Error bars are shown in mean+/- S.E.M. Statistical significance was tested using one-way analysis of variance (ANOVA) with Tukey's multiple comparison test. ns, non-significant; *, p<0.05; **, p< 0.01; ***, p< 0.001; ****, p< 0.0001

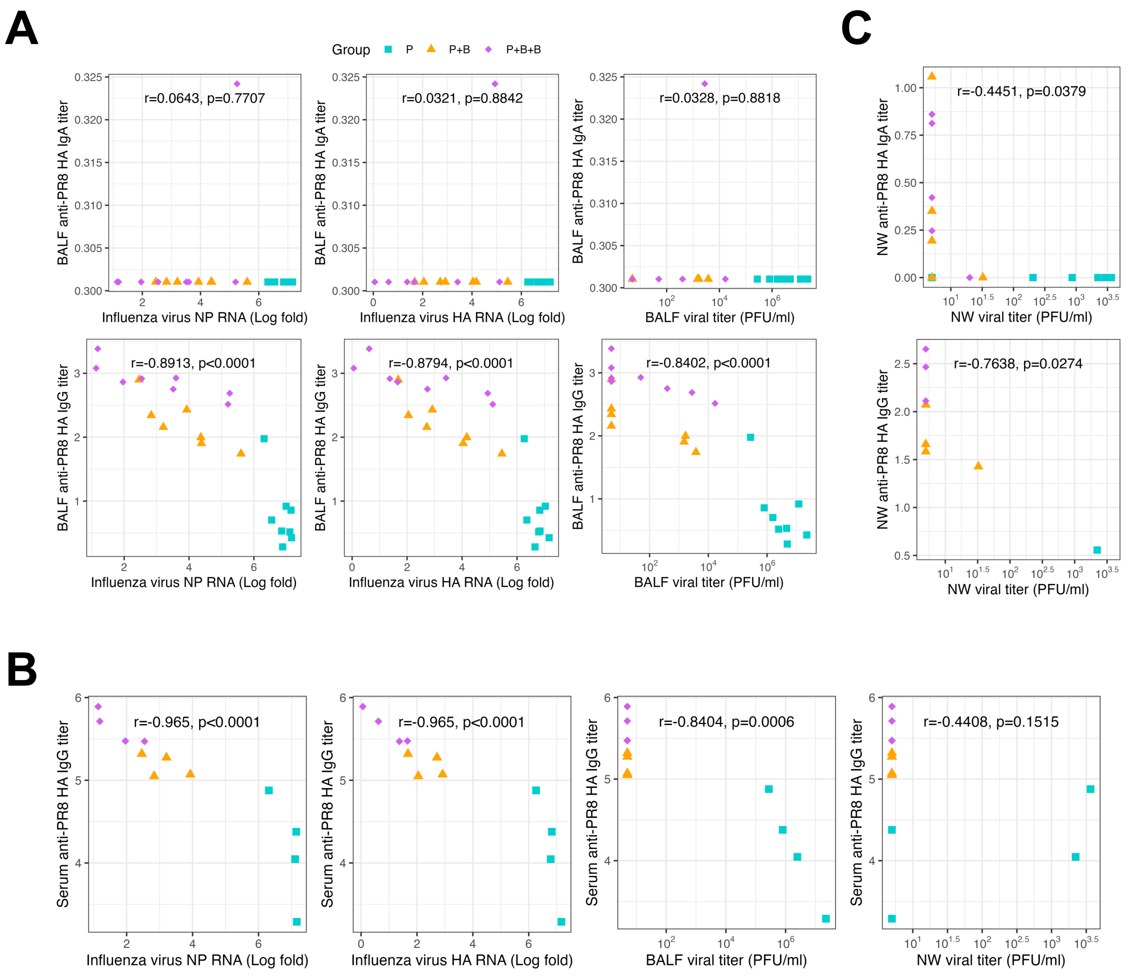


**Fig. S4. Mucosal IgG correlate negatively with viral burden**
(A to C) Balb/c mice were immunized with one, two or three doses of 0.05μg of PR8 HA mRNA-LNP intramuscularly (P, P+B, or P+B+B). Six weeks after the second boost, mice were challenged with $10^4$ PFU of A/PR8. Nasal wash, BALF, serum, and lung tissues were collected 3 days after infection. Infectious viral titers, viral RNA levels, and PR8 HA-specific antibody responses were determined. Spearman correlations denoted by R between anti-HA antibody titers and either viral titers or viral RNA levels in BALF (A), serum (B), and nasal wash (C). Statistical significance of correlations was assessed using two-tailed t-test. P-values of <0.05 were considered statistically significant.

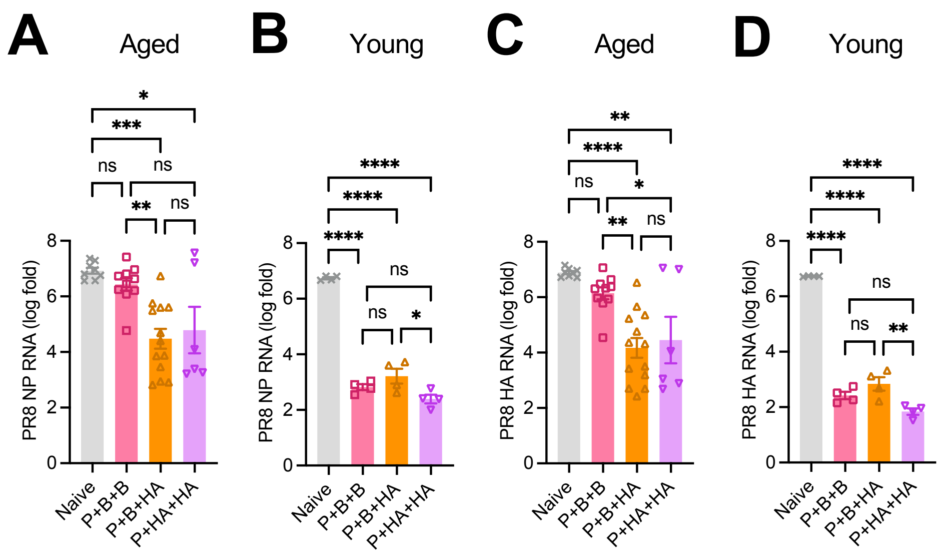


**Fig. S5. Intranasal HA booster reduces lung viral RNA load in aged mice**
(A to D) Aged Balb/c mice were primed and boosted with 0.05μg of PR8 HA mRNA-LNP intramuscularly and received either an additional dose of PR8 HA mRNA-LNP (Prime and boost and boost; P+B+B) or 5μg recombinant PR8 HA protein intranasally (Prime and boost and HA; P+B+HA). Another group of aged mice were primed with 0.05μg of PR8 HA mRNA-LNP intramuscularly and received two doses of 5μg recombinant PR8 HA booster intranasally (Prime and HA and HA; P+HA+HA). A paired young cohort was prepared. Six weeks after the second boost, mice were challenged with $10^4$ PFU of A/PR8. Lung tissues were collected 3 days after infection. Viral RNA levels were determined by RT-qPCR using primers targeting PR8 NP gene (A and B) and PR8 HA gene (C and D). Error bars are shown in mean+/- S.E.M. Statistical significance was tested using one-way analysis of variance (ANOVA) with Tukey's multiple comparison test. ns, non-significant; *, $p<0.05$; **, $p< 0.01$; ***, $p< 0.001$; ****, $p< 0.0001$

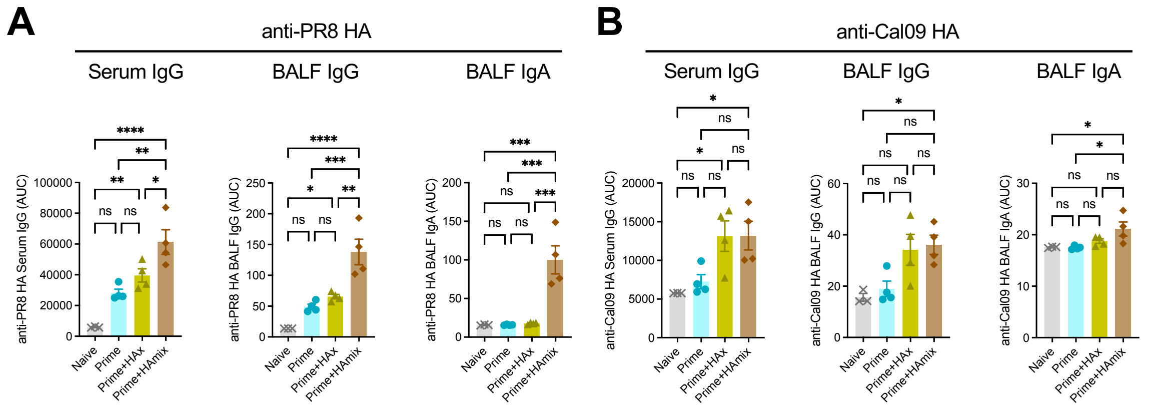


**Fig. S6. Multivalent intranasal HA booster expands the breadth of humoral immunity**
(A and B) Balb/c mice were primed with 1μg of PR8 HA mRNA-LNP intramuscularly and boosted intranasally with 5μg of heterologous Cal09 HA protein (Prime+HAx), recombinant HA cocktail (1.67μg each of PR8, Cal09, VN04 HA) (Prime+HAmix), or remained untreated (Prime). Six weeks after the boost, serum and BALF were collected. PR8 HA-specific (A) or Cal09 HA-specific (B) IgG and IgA responses were measured by ELISA. Error bars are shown in mean+/- S.E.M. Statistical significance was tested using one-way analysis of variance (ANOVA) with Tukey's multiple comparison test. ns, non-significant; *, p<0.05; **, p< 0.01; ***, p< 0.001; ****, p< 0.0001

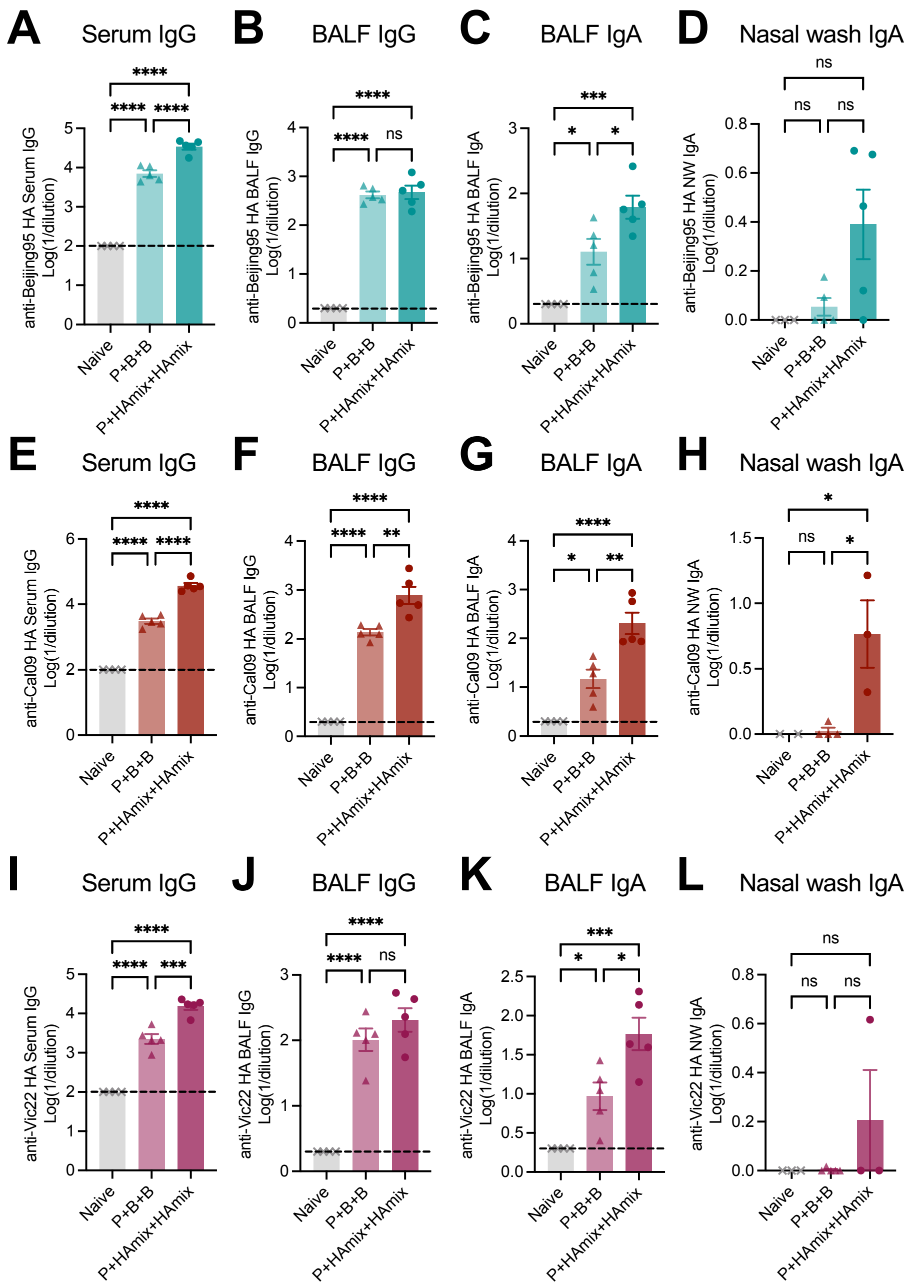


**Fig. S7. Bivalent IN HA booster induces systemic and mucosal antibodies against heterologous H1 strains**

(A to L) Balb/c mice were primed with 0.05µg of PR8 HA mRNA-LNP intramuscularly and boosted twice with recombinant HA cocktail (2.5µg each of PR8 and MI15 HA) intranasally (Prime and HA mix and HA mix; P+HAm+HAm) or received three doses of PR8 HA mRNA-LNP intramuscularly (Prime and boost and boost; P+B+B). Six weeks after the second boost, mice were challenged with 5000 PFU of MI15. On day 6, serum, nasal wash, and BALF were collected. (A to D) A/Beijing/262/1995 (H1N1; Beijing95) HA-specific, (E to H) A/California/04/09 (H1N1; Cal09) HA-specific, and (I to L) A/Victoria/4897/2022 (H1N1; Vic22) HA-specific systemic and mucosal IgG and IgA responses were measured by ELISA. Error bars are shown in mean+/- S.E.M. Statistical significance was tested using one-way analysis of variance (ANOVA) with Tukey's multiple comparison test. ns, non-significant; *, p<0.05; **, p< 0.01; ***, p< 0.001; ****, p< 0.0001
